# The Molecular Characterization of Hepatitis A Virus Strains Circulating during Hepatitis A Outbreaks in São Paulo, Brazil, from September 2017 to May 2019

**DOI:** 10.3390/v14010073

**Published:** 2021-12-31

**Authors:** Samira Chuffi, Michele S. Gomes-Gouvêa, Luciana V. B. Casadio, Ana Catharina S. S. Nastri, Mario P. Gonzalez, André L. F. Cotia, Amanda G. D. Aranda, Simone B. Tenore, Suzane K. Ono, Fernanda M. Malta, Geraldine Madalosso, Paulo R. A. Ferreira, Flair J. Carrilho, João R. R. Pinho

**Affiliations:** 1 Laboratório de Gastroenterologia e Hepatologia Tropical—LIM/07, Departamento de Gastroenterologia, Instituto de Medicina Tropical, Faculdade de Medicina da Universidade de São Paulo, Universidade de São Paulo, Av. Dr. Enéas Carvalho de Aguiar, 500, São Paulo 05403-000, SP, Brazil; sa.chuffi@gmail.com (S.C.); joao.pinho@einstein.br (J.R.R.P.); 2 Hospital das Clínicas, Departamento de Moléstias Infecciosas e Parasitárias, Faculdade de Medicina da Universidade de São Paulo, São Paulo 05403-000, SP, Brazil; luciana.vilasboas@gmail.com (L.V.B.C.); ana.cssn@gmail.com (A.C.S.S.N.); 3 Instituto de Infectologia Emilio Ribas, São Paulo 05403-000, SP, Brazil; mpgonzalez@terra.com.br (M.P.G.); andre_cotia_@hotmail.com (A.L.F.C.); amanda.dena.aranda@gmail.com (A.G.D.A.); 4 Centro de Referência e Treinamento—CRT DST-AIDS, São Paulo 05403-000, SP, Brazil; simone.tenore@crt.saude.sp.gov.br; 5 Hospital São Paulo, Disciplina de Infectologia, Escola Paulista de Medicina, Universidade Federal de São Paulo, São Paulo 05403-000, SP, Brazil; paulo.abrao.ferreira@gmail.com; 6 Hospital das Clínicas, Departamento de Gastroenterologia, Faculdade de Medicina da Universidade de São Paulo, São Paulo 05403-000, SP, Brazil; skon@usp.br (S.K.O.); fjcarril@usp.br (F.J.C.); 7 Laboratório de Técnicas Especiais, Hospital Israelita Albert Einstein, Albert Einstein Medicina Diagnóstica, São Paulo 05403-000, SP, Brazil; fernanda.malta@einstein.br; 8 Epidemiological Surveillance Center, Disease Control Coordination, State of São Paulo Department of Health, São Paulo 05403-000, SP, Brazil; gmadalosso@gmail.com

**Keywords:** hepatitis A virus, molecular epidemiology, outbreak

## Abstract

Outbreaks of hepatitis A may occur in countries of medium and high socioeconomic levels in which the population generally exhibits an increased susceptibility in young adults to this infection if they are not vaccinated against the hepatitis A virus (HAV). In Europe, an outbreak involved approximately 22 European countries with 4475 cases reported from 2016 to 2018; most of them were men who have sex with men (MSM). This outbreak expanded to North and South America, including Brazil, particularly in São Paulo city with 1547 reported cases from 2016 to 2019. In the present study, we characterized the HAV strains involved in the acute hepatitis A cases identified in the reference centers of São Paulo city during this outbreak. A total of 51 cases with positive anti-HAV IgM were included, 80.4% male, 68.6% of them between 20 and 40 years old and 41.7% MSM. HAV RNA was detected in 92% (47/51) of the cases. Subgenotype IA of HAV was identified and most of the strains were closely related to that isolated in outbreaks that occurred in different European countries in 2016. These results showed the epidemiological relation between these outbreaks and reinforce the need to implement vaccination against hepatitis A for the adult population, particularly for a population with a high-risk behavior.

## 1. Introduction

Outbreaks of hepatitis A are often seen in non-endemic regions whereas in endemic places they are uncommon because the majority of the population contracts the infection as a child and becomes immune [1]. Countries of medium and high socioeconomic power have suffered this paradoxical effect with an increased susceptibility of young adults to infection, generating more frequent outbreaks and often presenting cases with a more severe clinical picture [2].

Generally, HAV transmission occurs via a fecal-oral route, through the ingestion of contaminated water and/or food or through direct contact from person to person. The chance of contamination by direct contact from person to person increases with the sharing of personal objects and with inappropriate hygiene practices in the presence of infected and susceptible individuals in the same environment such as domestic environments and daycare centers [3,4,5]. Although fecal-oral transmission occurs through the aforementioned exposures, it can additionally be transmitted by unprotected sexual practices via direct contact with sites contaminated by feces, especially through the practice of oral-anal sex [6,7]. The particles of HAV are stable and resistant to room temperature and low pH conditions that favor its transmission via the fecal-oral route. Before symptomatic disease, the incubation period is usually approximately 28 days with viremia and viral elimination in feces during the first weeks after infection explaining the high probability of contamination in individuals who practice oral-anal sex [3,8].

Although outbreaks are frequent in regions of very low, low and intermediate prevalence for HAV, an expansion of the infection in 22 European countries was reported in 2016 that subsequently spread to North and South America. An epidemiological study published by the European Center for Disease Prevention and Control (ECDC) reported 4475 cases until September 2018, predominantly among the men who have sex with men (MSM) population [9,10,11,12]. The outbreak that started in Europe was first observed during the EuroPride event in Amsterdam, the capital of The Netherlands, and had participants from several countries with different sexual orientations such as heterosexuals, homosexuals, bisexuals and MSM. The molecular characterization of the HAV strains involved in these cases identified only subgenotype IA [9,10,11]. This outbreak spread to other countries and it was also noticed in Brazil, particularly in the city of São Paulo. According to the epidemiological bulletins published by the Health Surveillance Coordination (COVISA), there were 1547 cases of hepatitis A from 2016 to October 2019 only in the municipality of São Paulo in the Southeast region, which constitutes an important commercial and touristic hub [13]. In the present study, we characterized the HAV strains involved in the acute hepatitis A cases identified in several reference Centers of São Paulo during this outbreak period by sequencing and a phylogenetic analysis.

## 2. Materials and Methods

### 2.1. Patients and Samples

Serum and plasma samples were obtained over the period of September 2017 to May 2019 through an observational, prospective multicenter study, which evaluated consecutive patients with clinical conditions suggestive of acute hepatitis. These patients came from different reference services in São Paulo city, Brazil: 1—Medical Emergence of Instituto de Infectologia Emílio Ribas; 2—Division of Infectious and Parasitic Diseases and Division of Gastroenterology, Hospital das Clínicas, FMUSP; and 3—STD/AIDS Reference and Training Center. In the present study, we analyzed the cases characterized as acute hepatitis A that were defined by the presence of IgM antibodies to HAV (IgM anti-HAV positive).

### 2.2. Epidemiological Data

Epidemiological data including age, sex, suspected source of infection, recent travels, possible mode of infection and sexual orientation were collected using a standardized questionnaire.

## 3. HAV RNA Detection and Genotyping

### 3.1. RNA Extraction

For the extraction of viral RNA, a QIAamp Viral RNA Mini Kit (Qiagen, Hilden, Germany) was used according to the manufacturer’s instructions. The extraction was performed from 140 μL of serum or plasma and the extracted RNA was eluted in 60 μL of an elution buffer.

### 3.2. Reverse Transcription and Amplification

The HAV RNA was amplified by nested or hemi-nested PCR using primers previously described [8]. A SuperScript™ III One Step RT-PCR System with a Platinum™ Taq DNA Polymerase (Invitrogen, Carlsbad, CA, USA) kit was used for the reverse transcription and the first round of amplification; the enzyme Platinum™ Taq DNA Polymerase (Invitrogen, Carlsbad, CA, USA) was used for the second round.

The first reaction for the detection of HAV RNA was a reverse transcription followed by a hemi-nested PCR that amplified a 267 base pair (bp) fragment covering the VP1/2A region of the HAV genome. Primers 2950F (5′-TTGTCTGTCACAGAACAATCAG-3′) and 3308R (5′-AGTCACACCTCTCCAGGAAAACTT-3′) were utilized for the reverse transcription and the first round of the amplification under the following thermal cycling conditions: reverse transcription at 50 °C for 30 min; activation of the Taq polymerase at 94 °C for 2 min; and 35 cycles at 94 °C for 15 s, 55 °C for 30 s and 68 °C for 30 s. For the second round of this hemi-nested PCR, the primers 2950F and 3217R (5′-AGGGGGTGGAAGTACTTCATTTGA-3′) were used with the following thermo cycling conditions: 94 °C for 2 min and 35 cycles at 94 °C for 15 s, 55 °C for 30 s and 72 °C for 30 s. All negative samples in this PCR reaction were also tested using a commercial real-time PCR assay RealStar^®^ HAV RT-PCR kit 1.0 (Altona Diagnostics GmbH, Hamburg, Germany) for the confirmation of the results.

All samples with detectable HAV RNA were submitted to the amplification of the complete VP1 region (953 bp fragment) of the HAV genome by a nested PCR using the primers 2167F (5′-GTTTTGCTCCTCTTTATCATGCTATG-3′) and 3308R for the reverse transcription and the first round and 2172F (5′-GCTCCTCTTTATCATGCTATGGAT-3′) and 3125R (5′-CCTGCATTCTATATGACTCT-3′) for the second round. The thermal cycling conditions of these reactions were: reverse transcription at 50 °C for 30 min; activation of the Taq polymerase at 94 °C for 2 min; and 35 cycles at 94 °C for 15 s, 45.9 °C for 30 s and 68 °C for 30 s (reverse transcription and first round); 94 °C for 2 min and 35 cycles at 94 °C for 15 s, 53.6 °C for 30 s and 72 °C for 30 s (second round).

### 3.3. Nucleic Acid Sequencing

The amplified fragments (267 bp of VP1/2A and 953 bp of the complete VP1 region) were purified using an ExoSAP-IT PCR Clean-up Kit (GE Healthcare, Little Chalfont, Buckinghamshire, UK) and used as template for sequencing with a BigDye Terminator version 3.1 Cycle Sequencing Kit (Applied Biosystems™, Foster City, CA, USA) and an automatized sequencer (3500 Genetic Analyzer—Applied Biosystems™, Foster City, CA, USA).

### 3.4. Genotype and Subgenotype Determination

For the genotype/subgenotype classification, the sequences of each region of all amplified samples were aligned using the ClustalW program integrated into BioEdit software version 7.0.8 [14] together with the reference sequences of the different genotypes/subgenotypes deposited in GenBank, including sequences isolated from recent hepatitis A outbreaks in the European Union. The phylogenetic analyses were conducted in MEGA X and the analysis was performed by a maximum likelihood method using a Tamura 3 parameter model (data set with VP1/2A 267 bp sequences) or GTR + G + I (data set with complete VP1 region sequences) as determined by the model selection analysis of MEGA X [15]. To estimate the robustness of the phylogenetic tree, a Bootstrap approach (1000 replicates) was used. The phylogenetic trees were visualized and edited with Figtree version 1.4.2. (http://tree.bio.ed.ac.uk/software/figtree/, accessed on 15 July 2020).

## 4. Results

### 4.1. Epidemiological Data

The demographic and epidemiological characteristics of the acute hepatitis A cases analyzed in this study are shown in Table 1.

During the study period, 51 cases that were characterized as acute hepatitis A (IgM anti-HAV positive) were included. Most individuals (80.4%; 41/51) were male with 29 years old as the median age (range 19–64). Age-stratified analyses showed that most of these individuals were in the age group of 20–30 years (51%; 26/51) followed by the age group of 31–40 years (35.3%; 18/51). Considering age stratified by sex, we observed that most of the cases (68.6%; 35/51) were male in the age group of 20–40 years.

The epidemiological data regarding sexual orientation, suspected source of infection, recent travels and possible mode of infection were collected from 24 cases that answered the questionnaire. From these cases, 7 (29.2%) were women and 17 (70.8%) were men. Most of them (58.3%; 14/24) declared they were heterosexuals and 41.7% (10/24) declared themselves to be men who have sex with men. A recent history (previous 6 months) of multiple partners (3 or more) was reported by 8 individuals (33.3%) and all individuals denied a recent use of intravenous drugs. Considering the period from 2 weeks to 3 months before the onset of symptoms, the majority (70.8%; 17/24) of the individuals had a history of recent travels, 58.3% (14/24) reported eating raw or undercooked seafood and 37.5% (9/24) drank water from a suspected source. Direct contact with a suspect or a confirmed hepatitis case was reported by 3 (12.5%) individuals and direct contact with someone from Europe was reported by 2 (8.3%).

Among the MSM group (10/24), the median age was 29 years (range 19–49); 7 reported that they had three partners within the previous 6 month period and 5 were also affected by other sexually transmitted infections [HIV (n = 2) or syphilis (n = 3)].

### 4.2. Detection of HAV RNA, Genotyping and Molecular Phylogenetic Analyses

The presence of viremia was detected in 47 of the 51 analyzed samples; in 45 of them, HAV RNA was detected by the amplification of the VP1-2A region using a hemi-nested RT-PCR methodology and in 2 by using the commercial real-time PCR assay RealStar^®^ HAV RT-PCR kit 1.0.

**Figure 1:**
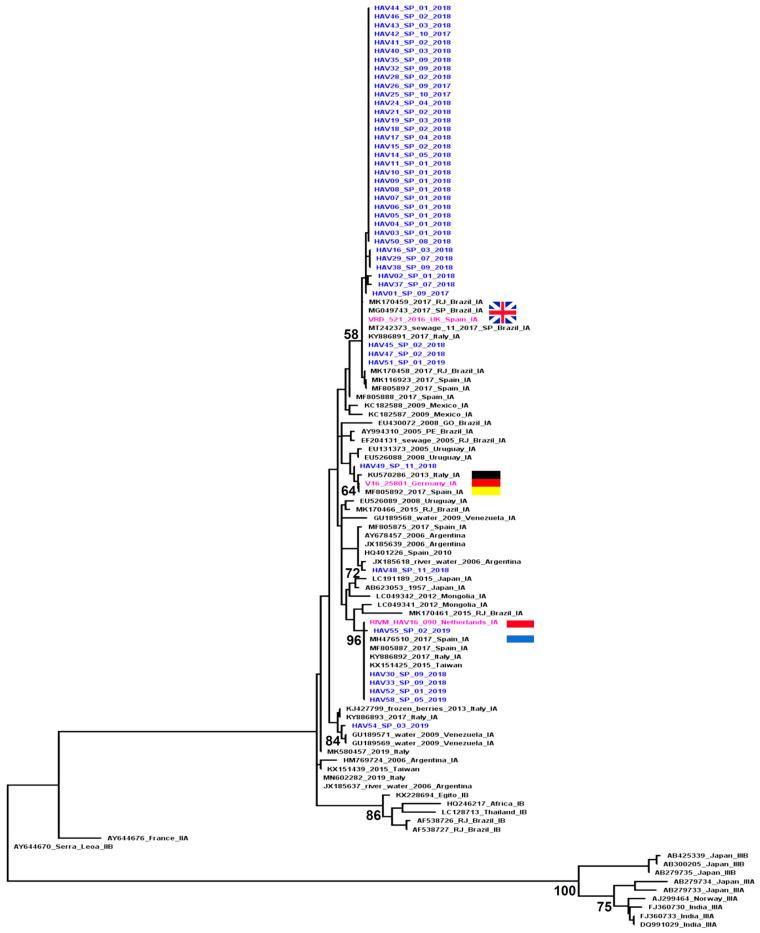
Phylogenetic tree of VP1/2A sequences (267 bp) from HAV strains isolated from acute hepatitis A cases identified in São Paulo city, Brazil, during 2016–2019. The analysis included 105 sequences: 45 from this study (in blue and identified with the sample number followed by the region, month and year of collection) and 60 reference sequences obtained from GenBank (identified with the accession number followed by the geographical origin and subgenotype). The reference clusters of HAV sequences isolated during the outbreak observed in European countries are highlighted in pink: VRD_521_2016 (United Kingdom), RIMV-HAV16-090 (Amsterdam, The Netherlands) and V16-25801 (Germany). Bootstrap values are shown along each main branch.

**Figure 2:**
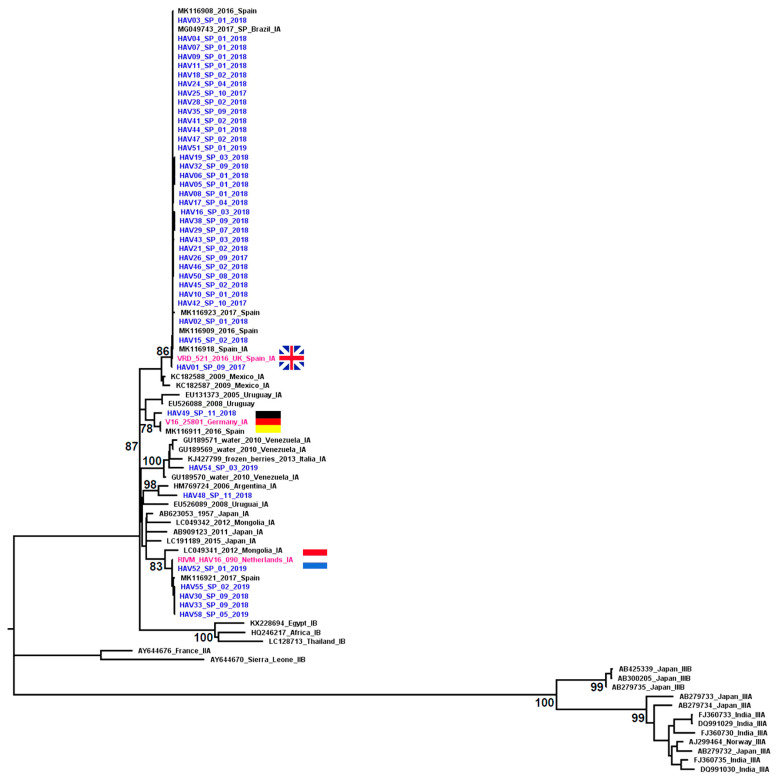
Phylogenetic tree of complete VP1 sequences (953 bp) from HAV strains isolated from acute hepatitis A cases identified in São Paulo city, Brazil, during 2016–2019. The analysis included 84 sequences: 42 from this study (in blue and identified with the sample number followed by the region, month and year of collection) and 42 reference sequences obtained from GenBank (identified with the accession number followed by the geographical origin and subgenotype). The reference clusters of HAV sequences isolated during the outbreak observed in European countries are highlighted in pink: VRD_521_2016 (United Kingdom), RIMV-HAV16-090 (Amsterdam, The Netherlands) and V16-25801 (Germany). Bootstrap values are shown along each main branch.

The complete VP1 region of the HAV genome was amplified from 42 of the 47 samples with HAV RNA detectable. The sequencing results were obtained from all samples amplified by the nested PCR: VP1-2A (267 bp) and VP1 (953 bp) regions were successfully sequenced from 42 samples and from 3 samples, only the smallest VP1-2A region was sequenced. Molecular phylogenetic analyses based on the two genomic regions characterized showed that all the sequences grouped with subgenotype IA; however, the HAV strains circulating in São Paulo (Brazil) during the study period were grouped into five distinct clusters inside the IA clade: 3 clusters were formed by strains related to the European HAV outbreaks and 2 had different origins (Figure 1 and Figure 2).

Most of the HAV strains identified in São Paulo were closely related to the strains implicated in the HAV outbreaks that occurred in different European countries in 2016. Of the 45 sequences from the VP1-2A region, 43 (95.5%) clustered together with strains identified in different European countries during the HAV outbreak in 2016: 37 clustered with the circulating strain in the UK (VRD_521_2016), five with the strain circulating in Holland (RIVM-HAV16-090) and one with the strain circulating in Germany (V16-25801) (Figure 1). The complete VP1 region was also characterized in 40 of these 43 samples and the results were in agreement with those obtained based on the VP1-2A region (Figure 2).

The sequences identified in São Paulo that grouped with the VRD_521_2016 cluster included cases identified in September 2017 (when the study began), throughout 2018 and in the first month of 2019. The sequences that grouped with the RIVM-HAV16-090 cluster included cases identified from September 2018 to May 2019 (end of case inclusion period of the study). The demographic characteristics of the individuals infected by the HAV strains that were grouped into these two clusters were similar to those described to complete the casuistic included in the study (Table 1). Epidemiological and risk factor data were available from 51.3% (n = 19/37) of the individuals infected by the HAV strains that grouped with the United Kingdom cluster (VRD_521_2016). The main risk factors observed among this group were: travel 2 months before the symptom onset (63%) (all trips were to regions within Brazil); raw or undercooked seafood consumption (52.6%); declared themselves to be MSM (42%); drinking water from an unsafe source (36.8%); and a recent history of multiple partners (36.8%). Only 2 of the 5 individuals with HAV strains related to The Netherlands cluster (RIVM-HAV16-090) had epidemiological data available: both were MSM with a recent history of multiple partners; had travelled 2 months before the symptom onset (one of them had traveled to regions within Brazil and other to the EUA); and had reported the consumption of raw or undercooked seafood. One of them reported contact with an infected person.

The sequence HAV49_SP_11_2018 collected at the end of 2018 was the only one that grouped with the cluster formed by the sequences isolated during the HAV outbreak in Germany (V16-25801). This strain was isolated from a 30 year old woman who reported international travel to Thailand in the previous month before the onset of symptoms.

Two sequences were grouped into clusters not related to those implicated in the European HAV outbreaks. The sequence HAV48_SP_11_2018 showed a greater similarity to the reference sequences isolated from a water sample and a human case in Argentina; this strain was isolated from a 64 year old man who reported traveling to several regions in northeastern Brazil and to Peru during the previous month before the onset of symptoms. The HAV54_SP_03_2019 sequence was grouped together with sequences isolated from a river water sample in Venezuela and was isolated from a 36 year old man, a Venezuelan immigrant who migrated to Brazil across the border in Rondônia (north region of the country) and presented the first symptoms during the trip whilst still on the Venezuelan side.

## 5. Discussion

In the present study, we analyzed cases of hepatitis A that occurred during an outbreak of the disease in the city of São Paulo, Brazil, including the epidemiological data and genetic characterization of the strains involved in this outbreak. The initial period (September 2017) for the inclusion of cases in this study took place approximately 1 year after the period in which the European hepatitis A outbreak began, which affected approximately 22 countries of the European Union [9,10,11]. It is known that this outbreak spread quickly, reaching countries in North and South America, specifically Brazil. It reached São Paulo at the end of 2016 with its peak during 2017, when half of the total number of the notified cases occurred only in the city of São Paulo (50.8%; 786/1547). According to the Health Surveillance Coordination (COVISA) of São Paulo city, during 2016, 2017, 2018 and 2019 (available data until 5 October 2019), 64, 786, 552 and 145 cases of acute hepatitis A were reported, respectively. Among these, the involvement of male individuals grew, representing 45% of those affected in 2016, 88% in 2017, 78% in 2018 and 77% by October 2019. There was an increasing involvement of individuals aged 18 to 39 years from 2017 onwards; furthermore, there was also an increase in the involvement of the sexual route in the transmission of the infection [13]. Published data on hepatitis A outbreaks in several European countries also report a similar profile [12].

Although the number of cases included in this study represents only a small portion of the extent of cases reported during the outbreak in the city of São Paulo (3.2%; 51/1547), the epidemiological characteristics observed were consistent with those described for the notified cases analyzed by COVISA [13]. In the present study, we also observed a higher frequency of male individuals (80.4%; 41/51) aged between 20 and 40 years. Of the cases from which we obtained information about sexual orientation, 41.7% (10/24) of the individuals were MSM, young people with a median age of 29 years old (19–49); 7 of them reported risky sexual behavior and 5 of them had other STIs. These data reinforce once again the similarity with the data described by COVISA considering the entire population of reported cases in São Paulo where 81.8% (1266/1547) of the affected cases were males with 32.5% (503/1547) of them contaminated by probable sexual acquisition [13].

Our data and those reported by COVISA including all cases reported in the city of São Paulo suggest a greater involvement of the sexual route in the spread of HAV during this outbreak. This was also reinforced by the finding of a recent study in Rio de Janeiro that described the HAV VRD_521_2016 strain infecting two MSM who reported sexual practices (unprotected anal-oral sex and multiple sexual partners) common among the cases identified in several HAV outbreaks [16]. According to data published by the ECDC (European Center for Disease Prevention and Control) regarding the outbreak of hepatitis A in European countries, there were 4475 confirmed cases up to September 2018, predominantly in the MSM population, although more precise data have not been released regarding the distribution of the epidemic by sex [9,10,11,12]. It is noteworthy that we could not rule out that there was a possible reluctance to admit sexual orientation in all studies, including this one, which may affect the estimate of HAV infection by sexual contact in this group and affecting its true extent.

The molecular characterization of HAV allows the identification of the transmission route, maps the origin of the infection (especially in outbreak investigations) and makes it possible to analyze the evolution of the virus at a molecular level [3,8].

The phylogenetic inference made in this study was based on two genomic regions (VP1-2A and VP1 complete) and showed similar results; however, we observed in the results obtained with the sequences of the VP1-2A region a lower bootstrap value supporting the HAV subgenotype clusters, especially subgenotype IA and IB clusters. Satisfactory and reliable results were obtained by analyzing the complete VP1 region, as described in previous studies that analyzed both genomic regions [8,17]. The characterization of a more comprehensive region such as the complete VP1 with approximately 953 bp was important to validate the results because it has more genetic information than the shorter VP1-2A region.

The phylogenetic analysis of the HAV sequences characterized in this study demonstrated the involvement of subgenotype IA in the outbreak that occurred in São Paulo city between 2017 and 2019. Five strains of the IA subgenotype were identified, three of them were closely related to the strains identified in the outbreaks in different European countries during the period from 2016 to 2018 (VRD_521_2016, RIMV-HAV16-090 and V16-25801), which represented most of the HAV sequences characterized in this study.

VRD_521_2016 was the only strain related to the European outbreak that was identified among the cases included in 2017 when the peak of the HAV outbreak in São Paulo occurred, which suggests that this was the strain responsible for triggering the outbreak in this region of Brazil. The results of the Bayesian analysis carried out in the aforementioned study that identified the VRD_521_2016 strain in Rio de Janeiro in September 2017 suggest that this strain was introduced into Brazil from Spain between the end of 2016 and the beginning of 2017, possibly related to the intense tourist movement promoted by the Olympic Games [16], which reinforces our hypothesis.

Based on our data, the RIVM-HAV16-090 strain was detected in São Paulo from September 2018 and its circulation was more restricted, probably due to the hepatitis A vaccination that was recommended in São Paulo city from May 2018 to people that reported sexual practices with oral-anal contact.

The strain V16-25801 was detected in a single case and the epidemiological data available suggest that this infection occurred outside Brazil during international travel.

HAV strains not related to the strains involved in the hepatitis A European outbreak were observed in two cases (HAV48_SP_11_2018 and HAV54_SP_03_2019). The demographic and epidemiological data collected explain the observed phylogenetic relationships: a recent trip to Peru was reported by case HAV48_SP_11_2018, whose sequence grouped with the reference sequences from Argentina (HM769724) and Uruguay (EU526089); and the Venezuelan nationality and recent migration to Brazil of the case from whom the sequence HAV54_SP_03_2019 was isolated, which grouped with a reference sequence isolated from contaminated water in Venezuela (GU189570). This individual had recently arrived in Brazil and presented the first clinical symptoms during the trip, making the hypothesis of contamination in his country of origin more convincing.

Our results showed the epidemiological relation between the outbreaks observed in São Paulo and the outbreaks that occurred in different European countries in 2016; moreover, the circulation of the HAV strains from other origins was also demonstrated. These data reinforce the role of interconnectedness between people such as international travel in the spread of HAV infections and show that despite sexual acquisition having been the focus of the recent HAV outbreak as well as the focus of this study, infections caused by strains of HAV other than those disseminated in the outbreak among the MSM population are constantly in circulation.

The increase of hepatitis A incidence, mainly among men, observed in Brazil from the end of 2016 was more intense in São Paulo; however, it was also observed in other cities from the southeast and south regions of Brazil [18,19]. These regions have a low hepatitis A endemicity and a higher proportion of young and susceptible adults [20]. Considering that hepatitis A can be more severe among this age group [21], it is important to emphasize the need to implement vaccination against hepatitis A for this population—particularly for groups with high-risk behavior—even in developing countries such as Brazil because they may have areas with different levels of endemicity and a large population of young adults susceptible to HAV infection.

## Figures and Tables

**Table 1:** Demographic and epidemiological characteristics of acute hepatitis A cases occurring in São Paulo city, Brazil, over the period of September 2017 to May 2019.

Characteristics	Total	Individuals with HAV Sequences in the United Kingdom (VRD_521_2016) Cluster	Individuals with HAV Sequences in The Netherlands (RIVM-HAV16-090) Cluster
	n (%)	n (%)	n (%)
**Demographic characteristics**	**51**	**37**	**5**
Age, median (range)	29 (19–64)	29 (19–57)	27 (25–37)
Sex			
Male	41 (80.4)	30 (81)	4 (80)
Female	10 (19.6)	7 (18.9)	1 (20)
**Epidemiological characteristics and risk factors**	**24**	**19**	**2**
Men who have sex with men	10 (41.7)	8 (42)	2 (100)
Recent history (at least a 6 month period) of multiple partners (3 or more)	8 (33.3)	7 (36.8)	2 (100)
Contact with infected individuals	3 (12.5)	2 (10.5)	1 (50)
Travelled in the 2 months before symptom onset	17 (70.8)	12 (63)	2 (100)
Consumed unsafe drinking water	9 (37.5)	7 (36.8)	0
Raw or undercooked seafood	14 (58.3)	10 (52.6)	2 (100)
Contact with people from Europe	2 (8.3)	2 (10.5)	0

## Data Availability

Not applicable.
